# Integrative processing of text and multiple maps in multimedia learning: an eye-tracking study

**DOI:** 10.3389/fpsyg.2025.1487439

**Published:** 2025-08-01

**Authors:** Aiko Morita, Izumi Fukuya

**Affiliations:** Graduate School of Humanities and Social Sciences, Hiroshima University, Hiroshima, Japan

**Keywords:** multimedia learning, eye-tracking, multiple maps, text, geography

## Abstract

The present study aims to improve understanding of how learners pay attention to the simultaneous presentation of text and multiple pictures in multimedia learning, focusing on the relationship between learners’ reading strategies and learning performance. Specifically, we focused on multiple thematic maps because they should be compared to each other to understand the causal relationships. Thirty-six university students read fictitious geographic learning materials consisting of text and five thematic maps, and completed retention and comprehension tests. Learners’ eye movements were recorded and their relationships with learning outcomes were examined. First, we found that fixation duration on maps was positively correlated with comprehension test scores. Second, the longer fixation duration of high-performance learners begin at an early stage of learning. Third, the gaze shift frequency between maps was positively correlated with comprehension test scores. This study demonstrates that the reading strategy of paying attention to maps and comparing them frequently is associated with higher geographic comprehension.

## 1 Introduction

Multimedia-learning studies have investigated the effects of adding visual information to verbal learning materials. Generally, research supports a multimedia effect whereby learners achieve higher learning performance through a combination of verbal and visual information, compared with learners who use verbal information alone (for a review, see Butcher, 2014; Carney and Levin, 2002; DeLeeuw and Mayer, 2008). However, only a few studies have focused on multiple picture references. Furthermore, while textbooks contain various types of visual information, such as diagrams, graphs, photographs, and charts (Mason et al., 2015), few studies have focused on maps in geography learning. The present study aims to improve understanding of how learners pay attention to the simultaneous presentation of text and multiple maps in multimedia learning, focusing on the relationship between learners’ reading strategies and learning performance.

### 1.1 Theoretical background of multimedia learning with text and pictures

Theories of multimedia learning, such as the cognitive theory of multimedia learning (CTML; Mayer, 2014) and the integrated model of text and picture comprehension (ITPC; Schnotz and Bannert, 2003; Schnotz, 2014), assume different but complementary contributions of text and pictures.

CTML posits the dual-channel assumption: it states that a working memory of limited capacity has an auditory–verbal and a visual pictorial channel. Processes of selection and organization result in a verbal mental model within the auditory–verbal channel and in a pictorial mental model within the visual–pictorial channel. These two mental models are integrated into a coherent mental representation. Limited capacity assumption states that each channel has limited capacity to hold the information. In addition, active processing assumption in CTML states that learning is an active process of collecting, organizing and integrating new information based on learner’s prior knowledge.

As another theoretical approach, ITPC emphasizes the representational differences between text and pictures. According to ITPC, text can provide more explicit guidance for constructing mental models than pictures, after scaffolding. Texts have predetermined processing orders, and are highly constrained. There is usually no predetermined processing order for pictures (Massironi, 2002; Sadoski and Paivio, 2001). Furthermore, the semantic content of pictures is usually less clearly defined and provides little conceptual guidance for comprehension. Studies have shown that learners spend more time reading text than pictures (Makransky et al., 2019). In contrast, pictures are expected to provide useful scaffolds in construction of an initial mental model (Schnotz and Wagner, 2018; Zhao et al., 2020). Learners can easily search for specific information and quickly grasp the outline of the mental model. This idea is supported by studies on the presentation order of texts and pictures (Eitel and Scheiter, 2015; Eitel et al., 2013; Gyselinck et al., 2008; Lindner et al., 2017; Schnotz and Bannert, 2003).

### 1.2 Multiple maps in multimedia learning

Among the various types of visual information, maps have particularly unique characteristics such as spatial properties of distance and direction. Kulhavy et al. (1993) posit that although maps are treated as simply one of many possible visuals, maps are granted a special status because they are visually processed as holistic images that preserve structural relationships and can be handled as single units in working memory, allowing simultaneous access to their information. Considering that pictures provide useful scaffolds for the construction of an initial mental model (Eitel and Scheiter, 2015; Eitel et al., 2013; Lindner et al., 2017), maps in which spatial relations correspond directly with the structure of the to-be-constructed mental model may be especially advantageous. Despite these features, map literacy has not been well developed thus far. Little is known about the higher-order cognitive processes involved in recognizing the entire content of a map in geographic problem solving (Wakabayashi, 2013).

Furthermore, some multimedia learning materials include multiple to-be-compared pictures. For example, it is common to present different types of thematic maps of a particular region in geographical learning materials (e.g., topographic maps, population maps, and land use maps). Learners are expected to compare not only text and maps but also multiple maps. The effectiveness of integrating multiple maps has been discussed in geography education research (Ardoin et al., 2014; Berglund, 2008; Cole, 2008; Dennis, 2006; Taylor and Hall, 2013; Travlou et al., 2008). In the case of learning with multiple to-be-compared pictures, learners must not only understand how information is encoded within each picture, but also how to relate the individual pictures to each other (Rexigel et al., 2024). However, reading strategies for materials containing text and multiple pictures have not been sufficiently studied. No explicit theoretical models are available to explain the cognitive processing of these complex combinations of external representations (Rexigel et al., 2024).

### 1.3 Reading strategy of text and multiple pictures

To investigate literacy or strategies of reading from text and pictures, we need to understand not only the learning outcome but also how students read text and pictures. Eye tracking technology has been used to investigate reading strategies.

Eye-tracking studies commonly use fixation duration and the number of fixations as key indices for analyzing attention allocation in a particular area. For example, eye-tracking studies of multimedia learning have revealed that learners sometimes pay little attention to pictures (Jian, 2017; Johnson and Mayer, 2012; Kombartzky et al., 2010; Stalbovs et al., 2015). Previous studies have shown that many learners use a text-driven strategy, and sometimes ignore information from the picture (Hannus and Hyönä, 1999; Hegarty, 1992; Holsanova et al., 2009; Hegarty and Just, 1993; Scheiter and Eitel, 2015).

More importantly, many studies have suggested that reading strategies vary according to learners’ learning abilities. Studies have shown that individuals with higher prior knowledge paid more attention to the relevant parts of a graphic than individuals with lower prior knowledge (Canham and Hegarty, 2010; Jarodzka et al., 2010).

Transition frequency has also been used as a measure of integration processes (Arndt et al., 2015; Hegarty and Just, 1993; Johnson and Mayer, 2012; Mason et al., 2013a; Ponce and Mayer, 2014; Rayner et al., 1978; Schnotz et al., 2014). Transition measures indicate the number of times a reader’s gaze shifts from a given area of verbal representation to a given area of graphical representation, and vice versa. Studies have shown that transitions between relevant text and pictures are positively correlated with learning performance scores (e.g., Kragten et al., 2015; Mason et al., 2013a; Mason et al., 2013b; Mason et al., 2015; Stalbovs et al., 2015). However, the transition between text and images is not sufficient to investigate reading strategies for text and multiple pictures. Transition among to-be-compared pictures should be investigated.

### 1.4 Overview of the present study

The purpose of this study was to investigate the relationship between learners’ eye movement patterns and learning outcomes when learning texts and the corresponding multiple maps. We measured fixation duration on text and maps to analyze when and to what extent learners paid attention to them. We also recorded the gaze shift between the text and multiple thematic maps. We measured the gaze shift not only between the text and map, but also among maps. We tested the following hypotheses:

*Hypothesis 1*: Total map fixation duration is hypothesized to be positively correlated with learning outcomes. As the active processing assumption in the CTML suggests, the fixation durations to the relevant parts are expected to be linked to learning performance (Canham and Hegarty, 2010; Jarodzka et al., 2010).

*Hypothesis 2*: High-performance learners fixate more on maps in the early stages of learning than low-performance learners. As the ITPC suggests, pictures are expected to provide useful scaffolds in an initial mental model construction (Schnotz and Wagner, 2018; Zhao et al., 2020). Considering that the spatial relations in a map directly correspond to the mental model (Eitel and Scheiter, 2015; Eitel et al., 2013; Lindner et al., 2017), maps are expected to be advantageous at the early stage.

*Hypothesis 3*: Map-to-map transition predicts learning outcomes better than map-to-text transition. The effectiveness of integrating multiple maps has been discussed in geography education research (Ardoin et al., 2014; Berglund, 2008; Cole, 2008; Dennis, 2006; Taylor and Hall, 2013; Travlou et al., 2008). Furthermore, as the ITPC suggests, learners first create a visual mental representation of the image and then construct a mental model.

## 2 Methods

### 2.1 Participants

The participants were 38 university students with normal or corrected-to-normal vision, who spoke Japanese as their native language. Before participating in the experiment, all participants read and signed a written informed consent form, explaining the purpose and procedure of the experiment and indicating that they could withdraw from participation at any time. Each participant received JPY 500 as compensation. Data from two participants were excluded because eye tracking did not work appropriately. A final sample of 36 students (20 female; mean age = 20.5; *SD* = 3.7) was included in the analyses.

### 2.2 Materials

#### 2.2.1 Learning materials

**Figure 1:**
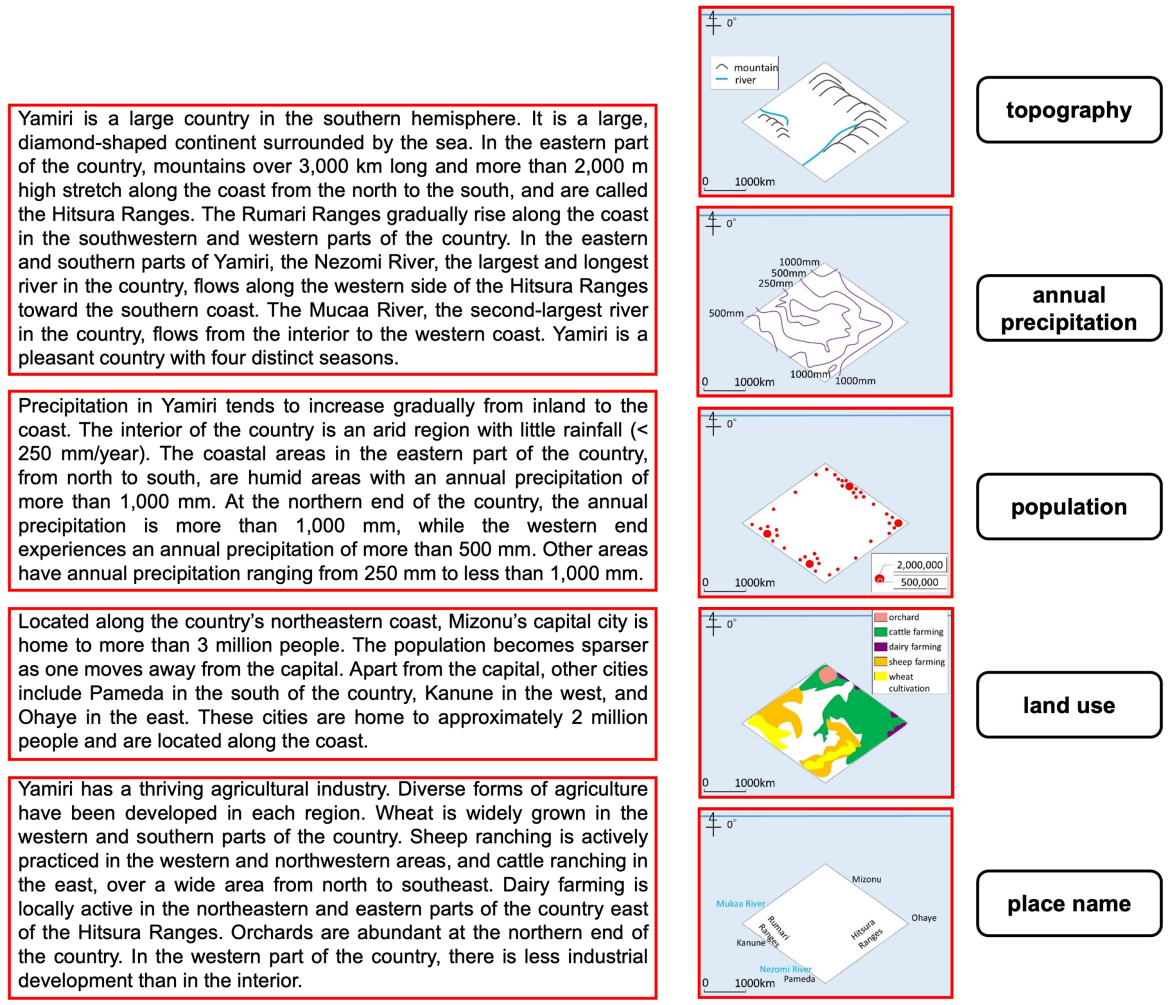
An example of the learning material (translated from Japanese). Red rectangles indicate the areas of interest (AOIs).

Geography education experts developed two learning materials for the experiment. The material was one page long (Figure 1). To address the prior knowledge effect, each material included geographic descriptions and maps of fictitious island, named “Yamiri” and “Kemuhi,” which have no meaning in Japanese. The learning material for “Yamiri” was modeled after the Australian continent, and the material for “Kemuhi” was modeled after the African continent. Each page contained a text of four paragraphs on the left side. Each paragraph described the topography, annual precipitation, population, and land use of the fictitious islands. The text was developed based on two types of Japanese junior high school geography textbooks. The paragraphs about “Yamiri” and “Kemuhi” were 798 and 791 Japanese characters long, respectively. On the right side, the page presented five thematic maps: topographical information (mountains and rivers), annual precipitation, population of the main cities (represented by a circle size), land use, and names of places. The five thematic maps were vertically aligned and evenly spaced, with their spatial distance from the text as illustrated in Figure 1. Notably, none of the participants recognized the real-world continent on which the fictitious learning materials were based.

#### 2.2.2 Retention and comprehension test

Each type of learning material was subjected to retention and comprehension tests with no time limit. The retention test consisted of six true-false questions (for example, “In the northern area of the country, the annual precipitation amounts to more than 1,000 mm”). The correct answers to each question could be found in the text and maps of the learning material. Participants were expected to memorize the facts they had read. Retention performance was calculated as points given for the sum of six questions (maximum score: 6 points).

The comprehension test consisted of six open-ended short-answer questions that required participants to make connections between different parts of the material. The questions were presented with the learning material. Participants were required to make inferences to generate their answers (for example, “This country has a small population. Explain why”). Each answer was scored between 0 and 2. Comprehension performance was calculated as the sum of the points received for answering the six questions (maximum score: 12 points).

Three geography education experts reviewed the test’s content validity. Specifically, they checked whether the comprehension questions appropriately targeted geographic causality. They also confirmed that the questions were neither too easy nor too difficult. The appropriateness of the difficulty level was further confirmed through a pilot study prior to the main experiment.

### 2.3 Apparatus

The learning materials and the tests were presented on a 23-inch monitor with a maximum resolution of 1,920 × 1,080 pixels, connected to a Tobii T60 eye tracker. The system recorded the eye movements at a sampling rate of 60 Hz. While learning the geography materials, raw gaze data were recorded using Tobii-Studio (2.2) software.

### 2.4 Eye movement measures

To analyze eye-movement data, we established AOIs (Areas of Interest). An AOI is a defined part of a presented stimulus in which fixations and saccades are analyzed separately (Holmqvist et al., 2011). Each of the four paragraphs and five maps were set as an AOI.

Two types of eye movement indicators were included in the analyses: (1) total gaze duration on the text and maps: The total gaze duration was the cumulative fixation time, including refixations, on the corresponding area of the screen. It was calculated for each minute (from 1 to 5 min) and reflected how much attention and cognitive investment learners devoted to the parts and how they changed during the learning time. (2) Number of transitions, which indicates the number of times a reader’s gaze shifts from one area to another. We counted the transitions between map and map, text (paragraph) and map, and text and text. The current study is particularly interested in map-to-map transitions.

### 2.5 Procedure

The participants were tested individually. They were informed beforehand that they would be required to read, understand, and learn some geographical learning material, and that a short test would follow. They were also informed that their eye movements would be recorded using an eye tracker system. The learning materials were presented on a computer screen. The participants were seated and their eyes were approximately 60 cm away from the monitor.

The experiment consisted of learning and testing sessions. In the learning session, one of the two materials was presented on the screen for 5 min. Participants were instructed to read the material silently. The eye movements of the participants were recorded during the session. During the testing session, the participants completed the retention and comprehension tests orally. The learning and testing sessions were repeated twice, using both types of learning material. The order of the materials was counterbalanced between the participants. Before the first learning session, we administered a practice trial consisting of learning and two types of tests.

### 2.6 Data analysis

We analyzed the relationship between the students’ eye movements and learning performance. First, we calculated the retention and comprehension test scores. On comparing the eye movements of high-and low-performance learners, we formed two groups according to their comprehension test scores. Second, we calculated the total gaze duration on the text and map areas during each minute of the learning session. Third, we calculated the gaze shift between map-to-map, map-to-text, and text-to-text. We analyzed whether these eye movement indicators were related to the test scores. In addition to a correlation analysis to determine the relationship between test scores and eye-movement indices, we compared high-and low-performance learners’ eye movements.

## 3 Results

### 3.1 Test performance and participant groups

The average score on the retention test was 4.46 (max = 6, *SD* = 0.95), and that on the comprehension test was 8.03 (max = 12, *SD* = 2.13). The correlation between the two tests was not significant (*r* = 0.295).

High and low performance was defined by the comprehension test scores. We used the scores because we specifically aimed to examine the contribution of maps to the understanding of geographic causality that require the integration of text and maps. The participants were divided into two groups based on the average comprehension score of 8.03. There were 18 high-performance learners with scores of 9.0 or more (average score = 9.72, *SD* = 0.89). The low-performance learners included 12 participants with scores of 7.0 or less (average score = 5.50, *SD* = 1.38). The *t*-test for the manipulation check revealed that the comprehension test scores differed significantly [*t* (28) = 10.190, *p* < 0.001, *d* = 3.695]. The average retention score of the high comprehension performance group was 4.71 (*SD* = 0.99) and that of the low-performance group was 4.06 (*SD* = 0.81). The *t*-test showed that the retention test scores were not significantly different between the groups [*t* (28) = 1.875, *p* = 0.071, *d* = 0.680].

### 3.2 Gaze duration on text and maps

In the following section, we analyze the relationship between the eye movement indices and test scores. Fixation durations of <80 ms were excluded from the analyses as readers were not presumed to have extracted any vital information during such short fixations (Rayner and Pollatsek, 1987).

To examine Hypothesis 1, we calculated the total gaze duration on the text and map areas during the 5-min learning session (Table 1). The correlation analysis between total gaze duration during the session and the test score indicated that the higher the comprehension test scores were, the longer the learners fixated on the maps (*r* = 0.559, *p* = 0.001). This relationship was not observed between comprehension test scores and gaze duration on the text (*r* = −0.055, *p* = 0.752). Furthermore, retention test scores were not related to gaze duration on either the text (*r* = 0.044, *p* = 0.797) or the map (*r* = 0.137, *p* = 0.425). We also conducted an exploratory analysis of fixation durations for each individual paragraph and map. However, since no systematic differences were observed across different types of text or map, these results are not reported in detail.

**Table 1:** Mean gaze durations (s) of the of high- and low-performance learners.

AOI	All participants		High-performance		Low-performance		Main effect
	Mean	(*SD*)	Mean	(*SD*)	Mean	(*SD*)	
Text							
Total	141.50	(33.22)	141.80	(34.09)	145.43	(35.38)	*ns*. *p* = .780
1 min	36.27	(7.05)	36.12	(7.47)	36.28	(7.46)	1, 2 min > 3, 4, 5 min *p* < .001
2 min	33.17	(5.68)	34.07	(5.13)	33.30	(6.87)	
3 min	25.78	(10.99)	26.07	(11.91)	26.01	(10.74)	
4 min	23.60	(10.03)	24.64	(11.19)	23.56	(8.33)	
5 min	22.69	(12.50)	20.89	(13.71)	26.28	(11.31)	
Map							
Total	77.57	(39.02)	91.12	(33.23)	49.01	(34.03)	High > Low *p* = .002
1 min	8.69	(6.31)	11.19	(6.28)	4.15	(3.99)	1, 2 min < 3, 4, 5 min *p* < .001
2 min	10.62	(5.87)	12.27	(4.95)	6.41	(3.91)	
3 min	18.12	(10.94)	20.23	(11.20)	12.21	(8.87)	
4 min	19.18	(11.67)	22.51	(11.41)	12.91	(10.52)	
5 min	20.96	(13.77)	24.92	(13.00)	13.34	(12.03)	

To examine Hypothesis 2, we calculated changes in gaze duration every minute (Table 1). Regarding the text area, a two-way ANOVA with performance (high/low) × time (1, 2, 3, 4, and 5 min) showed that the main effect of time was significant [*F*_(4, 112)_ = 15.615, *p* < 0.001, $\eta_{p}^{2}$ = 0.358]. Holm’s multiple analysis indicated that the participants fixated on the text longer during the 1st and 2nd minutes than during the 3rd, 4th, and 5th minutes. The main effect of performance group [*F*_(1, 28)_ = 0.079, *p* = 0.780, $\eta_{p}^{2}$ = 0.003], and the interaction [*F*_(4, 112)_ = 0.814, *p* = 0.504, $\eta_{p}^{2}$ = 0.028] were not significant. The results suggest that, regardless of the group, readers tended to pay more attention to the text during the first 2 min than during the later 3 min. Regarding gaze duration on the map areas, a two-way ANOVA showed that the main effect of group was significant [*F*_(1, 28)_ = 11.341, *p* = 0.002, $\eta_{p}^{2}$ = 0.288]. The high-performance group fixated on the maps longer than the low-performance group. The main effect of time was also significant [*F*_(4, 112)_ = 14.306, *p* < 0.001, $\eta_{p}^{2}$ = 0.338]. Holm’s multiple analysis indicated that the participants fixated on the maps longer during the 3rd, 4th, and 5th minutes than during the 1st and 2nd minutes. The interaction was not significant [*F*_(4, 112)_ = 0.670, *p* = 0.590, $\eta_{p}^{2}$ = 0.023].

In summary, the results suggest that readers tended to pay more attention to the text first and then to the maps, regardless of the group. More importantly, however, learners with high comprehension paid more attention to the map from the beginning than learners with low comprehension.

### 3.3 Transition among text and maps

To examine Hypothesis 3, we calculated the average number of three types of transitions: map-to-map, map-to-text, and text-to-text (Table 2). Correlation analysis indicated that the higher the comprehension test score, the more map-to-map transitions occurred (*r* = 0.452, *p* = 0.006). However, this relationship was not observed between the comprehension score and the map-to-text (*r* = 0.125, *p* = 0.467) or text-to-text transitions (*r* = −0.198, *p* = 0.246). No relationship was shown between retention test scores and transitions (*r* = 0.158, *p* = 0.359; *r* = 0.232, *p* = 0.174; *r* = 0.007, *p* = 0.968, respectively). Group comparison supported these results. The *t*-test showed that the high-performance group made more map-to-map transitions than the low-performance group [*t* (28) = 2.600, *p* = 0.015, *d* = 0.943]. There was no significant difference between the groups in map-to-text transition [*t* (28) = 1.279, *p* = 0.211, *d* = 0.464] or text-to-text transition [*t* (28) = −0.598, *p* = 0.555, *d* = −0.217]. In addition, we examined whether the type of map affected transition patterns. For map-to-text transitions, transitions between maps and topically matching text paragraphs (*M* = 35.75, *SD* = 20.64) were significantly more frequent than those involving mismatching topics (*M* = 28.61, *SD* = 11.87), although no significant association was found with comprehension scores. For map-to-map transitions, the precipitation, population, and land-use maps were referenced more frequently, whereas the map of place names was less frequently referenced. However, these transition patterns did not correlate with comprehension performance.

**Table 2:** Average number of transitions for high-and low-performance learners.

Type	All participants		High-performance		Low-performance		Main effect of performance
	Mean	(*SD*)	Mean	(*SD*)	Mean	(*SD*)	
Map-map	63.25	(48.15)	77.39	(54.47)	33.50	(25.25)	High > Low *p* = 0.015
Map-text	64.36	(25.75)	69.94	(23.50)	57.00	(32.01)	*ns*. *p* = 0.211
Text-text	11.69	(7.59)	11.44	(7.52)	13.25	(8.93)	*ns*. *p* = 0.555

## 4 Discussion

In this study, we attempted to extend current research on multimedia learning using text and pictures to the case of text and multiple to-be-compared pictures. Specifically, we focused on multiple thematic maps. We investigated whether learners’ reading strategies for text and maps were related to their learning outcomes.

### 4.1 Reading strategies for reading multiple maps

First, we examined the extent to which learners paid attention to multiple maps containing crucial spatial information. The results of gaze duration on text and maps throughout the learning session showed that the maps received more attention from high-performance learners than low-performance learners. This association was not found with regard to the text. Thus, Hypothesis 1 was supported. Although a causal relationship could not be established in this study, maps play a central role in understanding geographical content. This is consistent with empirical studies that suggest a relationship between high learning performance and paying attention to relevant pictures (Canham and Hegarty, 2010; Hannus and Hyönä, 1999; Jarodzka et al., 2010). We replicated the association in the case of multiple to-be-compared maps.

Second, learners’ attentional changes during a 5-min learning session were examined. It is assumed that the spatial relations between objects in a picture are mapped onto the corresponding semantic relations to form an initial mental model (Schnotz and Bannert, 2003). This is particularly true in the case of maps, in which the spatial relations directly correspond with the mental model. High-performance learners paid more attention to maps in the early stage of the learning session than low-performance learners. This difference seems to be consistent with Hypothesis 2. The reading strategy of paying attention to maps during the early learning stages may be related to better learning outcomes. Previous studies have assumed pictures provide useful scaffolds for the initial mental model construction (Schnotz and Wagner, 2018; Zhao et al., 2020). More importantly, however, this difference in strategy was also observed in the later learning stages. We did not observe the expected significant interaction between performance and time. Given that the effect size was also very small, and the mean values showed no clear pattern of interaction, this result suggests that the learning phase had a similar influence on both performance groups. Hypothesis 2 was not fully supported. Furthermore, it was found that both high-and low-performance learners paid more attention to the text than to the maps in the first 2 min, and increasingly shifted their attention to the maps as the learning session progressed. These results confirm the assumption that text and pictures have different functions for different purposes. Text provides explicit conceptual guidance (Schnotz and Wagner, 2018). Specifically, the text guides learners’ conceptual analysis of the subject matter, and learners engage in the process of coherence formation, which results in the formation of an initial mental model. These assumptions are supported by some previous studies that have shown that many learners use a text-driven strategy (Hegarty, 1992; Holsanova et al., 2009). The learners’ attentional changes in the present study show that learners do not use pictures as the dominant guidance but rather as scaffolds for constructing an initial mental model even when the pictures are maps.

The third and most noteworthy outcome of this study is that it demonstrates how learners pay attention to multiple pictures. The focus must be on not only the amount of attention but also the transition. The results showed that high-performance learners made map-to-map transitions more frequently than low-performance learners. Hypothesis 3 was supported. Map-to-map transition was shown to be critical for authentic geographic understanding. These results are consistent with the findings of previous map-based studies that have demonstrated the effectiveness of integrating multiple maps (Ardoin et al., 2014; Berglund, 2008; Cole, 2008; Dennis, 2006; Taylor and Hall, 2013; Travlou et al., 2008). Paying attention to the map has been suggested to support the understanding of geographic causality (von Reumont and Budke, 2020). In contrast, the present study suggests that map-to-text integration alone may not be sufficient for understanding geographic causality. These findings are inconsistent with previous studies that have suggested integration between text and maps is critical in multimedia learning (Mason et al., 2013a; Mason et al., 2013b; Mason et al., 2015; Stalbovs et al., 2015). These results suggest that map-based processing may play a particularly important role in the understanding of geographic causality.

In summary, our study extends multimedia learning research in two ways. First, while maps did not receive much attention in the eye tracking study, we clearly demonstrated that the reading strategy of paying attention to thematic maps is associated with geographic learning performance. Second, learners’ reading strategies for map-to-map integration are related to learning outcomes. We provide evidence of the contribution of picture-to-picture integration to multimedia learning.

The results provide guidance for designing written learning materials. Accordingly, designers of geography learning materials should emphasize maps and map-to-map relationships in the early stages of learning. This suggestion is consistent with instructions for other multimedia learning materials. Research has shown that learners sometimes pay little attention to visuals and even ignore them (Jian, 2016, 2017). Excessive dependence on text prevents learners from taking advantage of the positive effects of visual representations.

### 4.2 Limitations and future directions

It should be noted that we could not establish a causal relationship between learning strategies and learning outcomes. Future research should analyze the differences in performance based on learners’ prior knowledge. If learners with more prior knowledge use a more map-driven strategy, this means that a learning strategy with multiple maps is determined by the learner’s expertise. In addition, the testing procedure may have affected learners’ behavior. Participants were aware in advance that comprehension of geographic causal relationships would be required. Furthermore, it remains to be seen whether these findings can be generalized to other learning materials. Picture-to-picture integration can also be essential in other learning areas. Future studies should explore whether these results can be applied to other multimedia materials. Finally, it should be noted that all participants in the current study were university students. Further research is needed to determine the generalizability of these findings to younger learners.

## Data Availability

The raw data supporting the conclusions of this article will be made available by the authors, without undue reservation.

## References

[ref1] Ardoin N. M. Castrechini S. Hofstedt M. K. (2014). Youth–community–university partnerships and sense of place: two case studies of youth participatory action research. Child. Geogr. 12, 479–496. doi: 10.1080/14733285.2013.827872

[ref2] Arndt J. Schüler A. Scheiter K. (2015). Text–picture integration: how delayed testing moderates recognition of pictorial information in multimedia learning. Appl. Cogn. Psychol. 29, 702–712. doi: 10.1002/acp.3154

[ref3] Berglund U. (2008). Using children’s GIS maps to influence town planning. Child. Youth Environ. 18, 110–132. doi: 10.1353/cye.2008.0004

[ref4] Butcher K. R. (2014). “The multimedia principle” in The Cambridge handbook of multimedia learning. ed. Mayer R. E.. 2nd ed (New York, NY: Cambridge University Press), 174–205.

[ref5] Canham M. Hegarty M. (2010). Effects of knowledge and display design on comprehension of complex graphics. Learn. Instr. 20, 155–166. doi: 10.1016/j.learninstruc.2009.02.014

[ref6] Carney R. N. Levin J. R. (2002). Pictorial illustrations still improve students’ learning from text. Educ. Psychol. Rev. 14, 5–26. doi: 10.1023/A:1013176309260

[ref7] Cole A. G. (2008). Mapping students’ lives: children’s geographies, teaching and learning. Educ. Forum 73, 20–32. doi: 10.1080/00131720802539572

[ref8] DeLeeuw K. E. Mayer R. E. (2008). A comparison of three measures of cognitive load: evidence for separable measures of intrinsic, extraneous, and germane load. J. Educ. Psychol. 100, 223–234. doi: 10.1037/0022-0663.100.1.223

[ref9001] Dennis Jr. (2006). Prospects for Qualitative GIS at the Intersection of Youth Development and Participatory Urban Planning. Environ. Plann. A. 38, 2039–2054.

[ref9] Eitel A. Scheiter K. (2015). Picture or text first? Explaining sequence effects when learning with pictures and text. Educ. Psychol. Rev. 27, 153–180. doi: 10.1007/s10648-014-9264-4

[ref10] Eitel A. Scheiter K. Schüler A. Nyström M. Holmqvist K. (2013). How a picture facilitates the process of learning from text: evidence for scaffolding. Learn. Instr. 28, 48–63. doi: 10.1016/j.learninstruc.2013.05.002

[ref11] Gyselinck V. Jamet E. Dubois V. (2008). The role of working memory components in multimedia comprehension. Appl. Cogn. Psychol. 22, 353–374. doi: 10.1002/acp.1411

[ref12] Hannus M. Hyönä J. (1999). Utilization of illustrations during learning of science textbook passages among low-and high-ability children. Contemp. Educ. Psychol. 24, 95–123. doi: 10.1006/ceps.1998.0987, PMID: 10072311

[ref13] Hegarty M. (1992). Mental animation: inferring motion from static displays of mechanical systems. J. Exp. Psychol. Learn. Mem. Cogn. 18, 1084–1102. doi: 10.1037/0278-7393.18.5.1084, PMID: 1402712

[ref14] Hegarty M. Just M. A. (1993). Constructing mental models of machines from text and diagrams. J. Mem. Lang. 32, 717–742. doi: 10.1006/jmla.1993.1036

[ref15] Holmqvist K. Nyström M. Andersson R. Dewhurst R. Jarodzka H. van de Weijer J. (2011). Eye tracking: A comprehensive guide to methods and measures. Oxford: Oxford University Press.

[ref16] Holsanova J. Holmberg N. Holmqvist K. (2009). Reading information graphics: the role of spatial contiguity and dual attentional guidance. Appl. Cogn. Psychol. 23, 1215–1226. doi: 10.1002/acp.1525

[ref17] Jarodzka H. Scheiter K. Gerjets P. Van Gog T. (2010). In the eyes of the beholder: how experts and novices interpret dynamic stimuli. Learn. Instr. 20, 146–154. doi: 10.1016/j.learninstruc.2009.02.019

[ref18] Jian Y. C. (2016). Fourth graders’ cognitive processes and learning strategies for reading illustrated biology texts: eye movement measurements. Read. Res. Quart. 51, 93–109. doi: 10.1002/rrq.125

[ref19] Jian Y. C. (2017). Eye-movement patterns and reader characteristics of students with good and poor performance when reading scientific text with diagrams. Read. Writ. 30, 1447–1472. doi: 10.1007/s11145-017-9732-6

[ref20] Johnson C. I. Mayer R. E. (2012). An eye movement analysis of the spatial contiguity effect in multimedia learning. J. Exp. Psychol. Appl. 18, 178–191. doi: 10.1037/a0026923, PMID: 22309059

[ref21] Kombartzky U. Ploetzner R. Schlag S. Metz B. (2010). Developing and evaluating a strategy for learning from animations. Learn. Instr. 20, 424–433. doi: 10.1016/j.learninstruc.2009.05.002

[ref22] Kragten M. Admiraal W. Rijlaarsdam G. (2015). Students’ learning activities while studying biological process diagrams. Int. J. Sci. Educ. 37, 1915–1937. doi: 10.1080/09500693.2015.1057775

[ref23] Kulhavy R. W. Stock W. A. Kealy W. A. (1993). How geographic maps increase recall of instructional text. Educ. Tech. Res. Dev. 41, 47–62. doi: 10.1007/BF02297511

[ref24] Lindner M. A. Eitel A. Strobel B. Köller O. (2017). Identifying processes underlying the multimedia effect in testing: an eye-movement analysis. Learn. Instr. 47, 91–102. doi: 10.1016/j.learninstruc.2016.10.007

[ref25] Makransky G. Terkildsen T. S. Mayer R. E. (2019). Role of subjective and objective measures of cognitive processing during learning in explaining the spatial contiguity effect. Learn. Instr. 61, 23–34. doi: 10.1016/j.learninstruc.2018.12.001

[ref26] Mason L. Pluchino P. Tornatora M. C. Ariasi N. (2013a). An eye-tracking study of learning from science text with concrete and abstract illustrations. J. Exp. Educ. 81, 356–384. doi: 10.1080/00220973.2012.727885

[ref27] Mason L. Tornatora M. C. Pluchino P. (2013b). Do fourth graders integrate text and picture in processing and learning from an illustrated science text? Evidence from eye-movement patterns. Comput. Educ. 60, 95–109. doi: 10.1016/j.compedu.2012.07.011

[ref28] Mason L. Tornatora M. C. Pluchino P. (2015). Integrative processing of verbal and graphical information during re-reading predicts learning from illustrated text: an eye-movement study. Read. Writ. 28, 851–872. doi: 10.1007/s11145-015-9552-5

[ref29] Massironi M. (2002). The psychology of graphic images: Seeing, drawing, communicating. Mahwah, NJ: Erlbaum.

[ref30] Mayer R. E. (2014). “Cognitive theory of multimedia learning” in The Cambridge handbook of multimedia learning. ed. Mayer R. E.. 2nd ed (New York, NY: Cambridge University Press).

[ref31] Ponce H. R. Mayer R. E. (2014). An eye movement analysis of highlighting and graphic organizer study aids for learning from expository text. Comput. Hum. Behav. 41, 21–32. doi: 10.1016/j.chb.2014.09.010

[ref32] Rayner K. McConkie G. W. Ehrlich S. (1978). Eye movements and integrating information across fixations. J. Exp. Psychol. Hum. Percept. Perform. 4, 529–544. doi: 10.1037//0096-1523.4.4.529, PMID: 722245

[ref33] Rayner K. Pollatsek A. (1987). “Eye movements in reading: a tutorial review” in Attention and performance XII: The psychology of reading. ed. Coltheart M. (London: Erlbaum), 327–362.

[ref34] Rexigel E. Kuhn J. Becker S. Malone S. (2024). The more the better? A systematic review and Meta-analysis of the benefits of more than two external representations in STEM education. Educ. Psychol. Rev. 36:124. doi: 10.1007/s10648-024-09958-y

[ref35] Sadoski M. Paivio A. (2001). Imagery and text: A dual coding theory of reading and writing. Mahwah, NJ: Erlbaum.

[ref36] Scheiter K. Eitel A. (2015). Signals foster multimedia learning by supporting integration of highlighted text and diagram elements. Learn. Instr. 36, 11–26. doi: 10.1016/j.learninstruc.2014.11.002

[ref37] Schnotz W. (2014). “Integrated model of text and picture comprehension” in The Cambridge handbook of multimedia learning. ed. Mayer R. E.. 2nd ed (Cambridge, UK: Cambridge University Press), 72–103.

[ref38] Schnotz W. Bannert M. (2003). Construction and interference in learning from multiple representation. Learn. Instr. 13, 141–156. doi: 10.1016/S0959-4752(02)00017-8

[ref39] Schnotz W. Ludewig U. Ullrich M. Horz H. McElvany N. Baumert J. (2014). Strategy shifts during learning from texts and pictures. J. Educ. Psychol. 106, 974–989. doi: 10.1037/a0037054

[ref40] Schnotz W. Wagner I. (2018). Construction and elaboration of mental models through strategic conjoint processing of text and pictures. J. Educ. Psychol. 110, 850–863. doi: 10.1037/edu0000246

[ref41] Stalbovs K. Scheiter K. Gerjets P. (2015). Implementation intentions during multimedia learning: using if-then plans to facilitate cognitive processing. Learn. Instr. 35, 1–15. doi: 10.1016/j.learninstruc.2014.09.002

[ref42] Taylor K. H. Hall R. (2013). Counter-mapping the neighborhood on bicycles: mobilizing youth to reimagine the city. Tech. Know. Learn. 18, 65–93. doi: 10.1007/s10758-013-9201-5

[ref43] Travlou P. Owens P. E. Thompson C. W. Maxwell L. (2008). Place mapping with teenagers: locating their territories and documenting their experience of the public realm. Child. Geogr. 6, 309–326. doi: 10.1080/14733280802184039

[ref44] von Reumont F. Budke A. (2020). Strategies for successful learning with geographical comics: an eye-tracking study with young learners. Educ. Sci. 10:293. doi: 10.3390/educsci10100293

[ref45] Wakabayashi Y. (2013). Role of geographic knowledge and spatial abilities in map reading process: implications for geospatial thinking. Geographical Reports Tokyo Metropolitan University 48, 37–46.

[ref46] Zhao F. Schnotz W. Wagner I. Gaschler R. (2020). Texts and pictures serve different functions in conjoint mental model construction and adaptation. Mem. Cogn. 48, 69–82. doi: 10.3758/s13421-019-00962-0, PMID: 31372846

